# P-425. Implementation of Urinalysis with Reflex to Urine Culture at a Pediatric Health System: A Diagnostic Stewardship Intervention

**DOI:** 10.1093/ofid/ofaf695.641

**Published:** 2026-01-11

**Authors:** Zachary M Most, Hayden Dutton, Mehgan Kidd, Michael Sebert, Laura Filkins

**Affiliations:** University of Texas at Southwestern Medical Center, Dallas, TX; Children's Health System Dallas, Dallas, Texas; University of Texas Southwestern Medical Center Dallas, Dallas, Texas; UT Southwestern Medical Center, Dallas, Texas; University of Texas Southwestern/Children's Health, Dallas, Texas

## Abstract

**Background:**

Asymptomatic bacteriuria usually does not require treatment and is a common reason for inappropriate antibiotic use. Urine reflex testing is a widely utilized diagnostic stewardship tool that reduces unnecessary urine cultures while maintaining ease of use for providers. There are few reports of the impact of urine reflex testing in a pediatric healthcare system.

**Figure 1:**
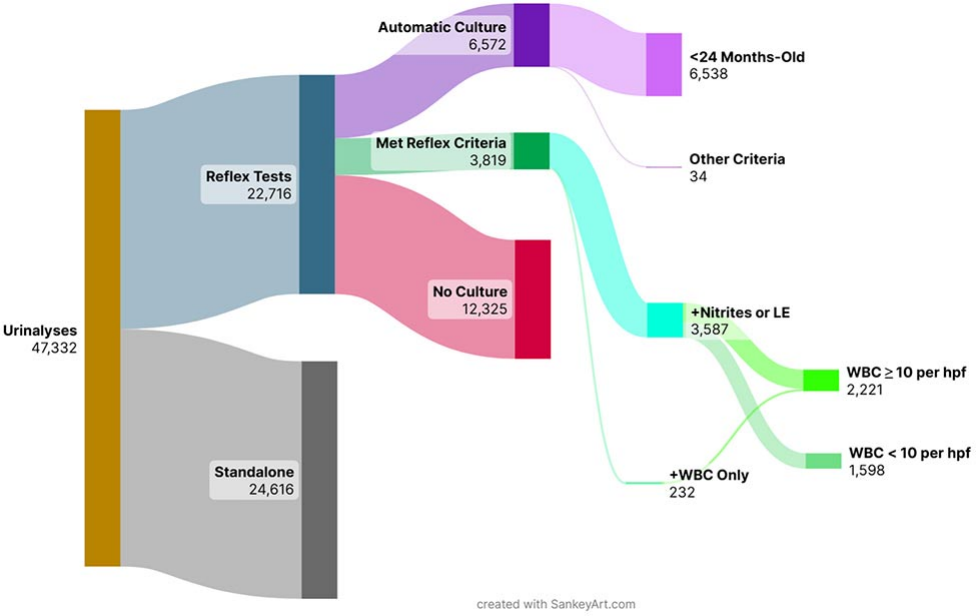
Breakdown of urine tests done after implementation of reflex testing LE - leukocyte esterase, WBC - white blood cells, hpf - high power field

**Figure 2:**
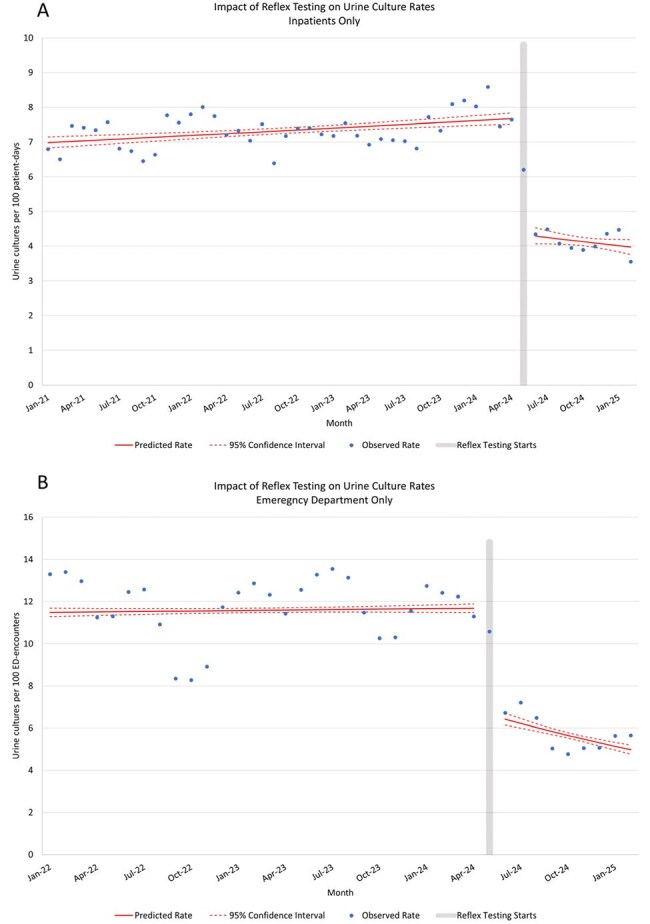
Change in urine culture rates after implementation of reflex testing. A) inpatient urine culture rate - the number of urine cultures collected on admitted patients per 100 patient-days. B) Emergency department urine culture rate - the umber of urine cultures collected on patients in the ED per 100 ED-encounters.

**Methods:**

Before implementing reflex testing, our healthcare system had a combined urinalysis and urine culture order that was widely used in the emergency department. In May 2024, we replaced the combination order with a “Urinalysis with Reflex to Urine Culture (suspected UTI)” order, which was made available to all providers. For this order, urine culture was only performed if reflex criteria were met. Our reflex criteria were at least one of: nitrites positive, leukocyte esterase small/medium/large, or urine WBC ≥ 10 per hpf. Patients under 24 months old were excluded from the reflex pathway and always had cultures done if this order was used. Providers could also select from a short list of conditions for which urine culture would be done regardless of urinalysis results. Urine culture utilization was compared between the pre-intervention period (January 2021 to May 2024) and post-intervention (May 2024 to February 2025) using interrupted time series analysis.

**Results:**

From May 23, 2024, to February 28, 2025, 22,716 urine reflex tests were ordered, of which 10,391 led to urine cultures and 12,325 (54.3%) did not reflex to culture (Figure 1). Patients who had urine cultures done were younger in the post-intervention period compared to the pre-intervention period (mean 4.1 years vs 6.2 years, P< 0.0001) and were less likely to have been tested in the emergency department (59.8% vs 66.6%, P< 0.001). Interrupted time series analysis demonstrated an immediate 44% decrease in urine culture incidence on inpatient units (95% CI 0.40-0.47, Figure 2a) and 43% decrease in urine culture incidence in the emergency department (95% CI 0.40–0.46, Figure 2b). This amounted to ∼1100 urine cultures avoided per month.

**Conclusion:**

Implementation of reflex urine culture testing in a pediatric healthcare system led to a large decrease in urine culture rate.

**Disclosures:**

Zachary M. Most, MD, MSc, VisualDx: Honoraria Laura Filkins, PhD, Avsana Labs: Advisor/Consultant|Biofire Diagnostics/Biomerieux: Grant/Research Support

